# Relationship Between Bioimpedance Vector Displacement and Renal Function After a Marathon in Non-elite Runners

**DOI:** 10.3389/fphys.2020.00352

**Published:** 2020-05-06

**Authors:** Lexa Nescolarde, Emma Roca, Paco Bogónez-Franco, José Hernández-Hermoso, Antoni Bayes-Genis, Jordi Ara

**Affiliations:** ^1^Department of Electronic Engineering, Universitat Politècnica de Catalunya, Barcelona, Spain; ^2^Department of Orthopedic Surgery and Traumatology, Hospital Universitari Germans Trias i Pujol, Barcelona, Spain; ^3^Department of Surgery, Faculty of Medicine, Universitat Autònoma de Barcelona, Campus UAB, Barcelona, Spain; ^4^Department of Cardiology, Hospital Universitari Germans Trias i Pujol, Barcelona, Spain; ^5^Department of Medicine, Faculty of Medicine, Universitat Autònoma de Barcelona, Barcelona, Spain; ^6^Research Program, Fundació Institut d’Investigació en Ciències de la Salut Germans Trias i Pujol, Barcelona, Spain; ^7^Department of Nephrology, Hospital Universitari Germans Trias i Pujol, Barcelona, Spain

**Keywords:** marathon race, BIVA, serum creatinine, C-reactive protein, serum creatine kinase, AKI stage 1

## Abstract

**Purpose:** This study investigates the relationship between whole-body bioimpedance vector displacement, using bioelectrical impedance vector analysis (BIVA), and renal function through serum biomarkers [creatinine, urea, sodium, C-reactive protein (CRP), and creatine kinase] and urine biomarkers after a marathon.

**Methods:** Bioimpedance measurements were taken among 19 non-elite runners at 24 h pre-race, immediately post-race, and at 48 h post-race. The bioimpedance measurements were analyzed by BIVA using the Hotelling’s T2 test. The runners were divided according to a cutoff of serum creatinine level immediately post-race in G1 (<1.2 mg/dl of serum creatinine level) and G2 (≥1.2 mg/dl of serum creatinine level). The increase of the serum creatinine levels in 83% of G2 runners was related to acute kidney injury (AKI) stage 1.

**Results:** Neither G1 nor G2 showed a creatinine clearance rate (CCr) lower than 60 ml/min. G2 showed a significant increase in CRP values at 48 h post-race *vs* baseline compared to G1 (*P* < 0.05), with over 5 mg/L (6.8–15.2) in 92% of the runners, and in CK values with over 215 U/L (282–1,882) at 48 h post-race in 100% of the runners. By BIVA, the 95% confidence ellipses of G2 showed shorter bioimpedance vectors than G1, with a noticeable minor Xc/H (*P* < 0.01), indicating an expansion on extracellular water and inflammation. The runners with 48 h post-race Xc/H values ≤30.5 Ω, with a decrease from −3 to −12% with respect to the Xc/H value at 24 h pre-race, indicated AKI stage 1 with 85.7% sensitivity and 91.7% specificity, with a direct correlation between AKI stage 1 with greater CRP values at 48 h post-race and bioimpedance vector displacement, but not with CK values at 48 h post-race.

**Conclusion:** Through this data collection, it was evidenced that a transient reduction in renal function is more related to inflammatory factors than muscle damage. The BIVA method along with serum biomarkers could be used to follow up the kidney function in runners.

## Introduction

The usual practice of sport has a direct positive impact on physical health in general (Hawley et al., 2014). In marathon races (42.195 km), non-elite participants increased by 25-fold in the last 40 years. For example, in the 2017 Barcelona marathon, there were 19,740 runners, whereas in the 2007 marathon there were 7,430 runners (Zurich Barcelona Marathon, 2019). However, marathon running can induce transient changes in renal filtration function due to neurohormonal, inflammatory, and oxidative stress factors or to hemodynamic influences on renal blood flow (McCullough et al., 2011; Hewing et al., 2015; Lipman et al., 2016). The glomerular filtration rate (GFR) provides the best index of overall kidney function, whereas serum creatinine is the most sensitive serum biomarker for detecting small changes in GFR (Dalton, 2010; Ferguson and Waikar, 2012). Acute kidney injury (AKI) was defined using the AKI Network (AKIN) criteria (Mehta et al., 2007). Stage 1 AKI was defined as a 1.5-fold to twofold increase or 0.3 mg/dl increase in serum creatinine from day 0 to peak creatinine value on either day 1 or day 2 (McCullough et al., 2011; Kellum et al., 2013). Other authors relate an acute kidney injury after contrast medium (CI-AKI) as an absolute increase ≥0.5 mg/dl over baseline serum creatinine (Maioli et al., 2012). The measurement of urea concentration, in blood or urine, is another indicator of kidney function similar to creatinine (Kratz et al., 2002; McCullough et al., 2011). Urea is the principal nitrogen waste product of protein metabolism, and it is eliminated from the body almost exclusively by the kidneys in urine, although serum creatinine is the main biomarker to define acute kidney injury (Kellum et al., 2013).

C-reactive protein (CRP) is one of the proteins involved in physical stress reactions, implicated in renal function acting as a risk factor for AKI and increasing in AKI (Tang et al., 2018). After a marathon, the CRP serum concentrations start to rise within 8 h after exercise and peak at 24 h after due to the inflammatory response caused by tissue injury. In non-elite marathon runners, CRP increases threefold at 24 h post-race, with this increase being even greater (40-fold) in ultraendurance races (Kim et al., 2009). CRP is considered as a biomarker of inflammation in AKI, impairing tubular regeneration and promoting fibrosis of injured renal tissue (Tang et al., 2018).

A strenuous, untrained exercise can damage muscle fibers, increasing membrane permeability and sarcomere degeneration. As a consequence, at 24 h post-race, large amounts of muscle proteins will be released into the bloodstream, like creatinine kinase (CK) (Brancaccio et al., 2007, 2010; Shin et al., 2016), which is easily assessed in the serum and is a sensitive, but not specific, marker of muscle tissue damage. Exertional (exercise-induced) rhabdomyolysis (ER) is well described in athletes and is the general term for muscle breakdown associated with strenuous exercise. In endurance exercise, there is no consensus about the CK level threshold, although healthy population CK values >15,000 U/L are predictive of renal impairment (Rawson et al., 2017). In marathon races, CK can increase threefold at 24 h post-race (Kim et al., 2009) without showing a correlation between AKI and muscle breakdown (Mansour et al., 2017). This is due to the fact that the marathon effort does not involve eccentric contractions, and rhabdomyolysis is also less common (Scalco et al., 2016). In ultra-endurance races, CK can increase 35-fold (Kim et al., 2009). Indeed after a 161-km ultramarathon, a CK of greater than 100,000 U/L has been frequently observed without apparent health consequences (Hoffman et al., 2012). Military recruits (Kenney et al., 2012) in basic training (*n* = 499) reported mean CK values of 734, 1,226, and 667 U/L on days 3, 7, and 14 post-exercise, respectively, and a maximum of 35,056 U/L; however, nobody developed clinical exertional rhabdomyolysis. The authors suggested that an increase of CK values by 50-fold above the normal limit can be a specific marker for exertional rhabdomyolysis in military recruits.

The bioelectrical impedance vector analysis (BIVA) (Piccoli et al., 1994) is a method to classify soft tissue mass and hydration based on patterns of direct bioimpedance measurements. Bioimpedance (Z) is measured in a whole-body configuration (standard bioimpedance measurement) (Foster and Lukaski, 1996) at 50 kHz using phase-sensitive devices. The components of the bioimpedance vector—resistance (R) and reactance (Xc)—are normalized by the height (H) of the subjects (R/H and Xc/H) and are represented in the resistance–reactance graph (RXc-graph). The vector measured on an individual is compared against the normal intervals of the reference population, expressed in percentiles of 50, 75, and 95% of normal (Gaussian) distribution in a bivariate, probabilistic plot (Morrison, 1967). The RXc-graph method allows (1) the evaluation of an individual vector: the displacements parallel to the major axis of tolerance ellipses indicating progressive changes of fluids, whereas displacements parallel to the minor axis are associated to variations of cell population density contained in soft tissues and different trajectories indicate combined changes in both fluid and soft tissue mass; (2) the evaluation of bioimpedance follow-up, by the trajectory of successive measurements of the impedance vector; and (3) the evaluation of groups of subjects using the bivariate 95% confidence ellipses of the mean vectors. Two mean vectors, from two independent groups of subjects, can be compared with the two-sample Hotelling’s T2 test (Hotelling, 1931). A mean vector displacement in one group of subjects can be evaluated with the paired one-sample Hotelling’s T2 test (Piccoli and Pastori, 2002). The main advantage of BIVA is that it does not require any assumption of soft tissue hydration (normal or abnormal) and body composition and is independent of body weight. The assessment of soft tissue is done through direct phase-sensitive bioimpedance measurement—without requiring the use of prediction equations, avoiding imprecision of individual estimations (> 10% variability) associated with regression equations (Piccoli et al., 1994; Lukaski et al., 2019). In addition, severe dehydration can occur before the appearance of clinical signs; however, these can be assessed by combining measurements of phase angle (PA) and Z vector length from phase-sensitive bioimpedance devices operating at 50 kHz and plotted on the RXc-graph (Lukaski et al., 2019).

The major clinical applications of BIVA are focused on kidney failure to adjust the ultrafiltration sessions (Piccoli, 1998, 2004; Pillon et al., 2004), to relate edema with mortality (Piccoli et al., 1996; Nescolarde et al., 2004; Piccoli, 2004; Bouchard et al., 2009), and more recently (Maioli et al., 2018) as a valid predictor of CI-AKI. The applications of BIVA method on endurance events are focused on relating fluid loss after a bout of exercise-induced dehydration with a lengthening of the impedance vector along the major axis of the tolerance ellipse compared to pre-trial measurements (Gatterer et al., 2014) and, with body mass changes concomitant with training and intensity of the exercise, finding a significant decrease in body mass and an increase in bioimpedance variables (Carrasco-Marginet et al., 2017; Giorgi et al., 2018).

Although previous studies have examined the effect of endurance exercise in the displacement of the bioimpedance vector, none of them has examined the relationship between the serum biomarkers of kidney function with bioimpedance measurements using BIVA. This study investigates, for the first time, the relationship between whole-body bioimpedance vector displacement by BIVA, using a 50-kHz phase-sensitive bioimpedance analyzer, with renal function through serum and urine biomarkers in non-elite marathon runners after a marathon.

## Materials and Methods

### Participants

Two months before the 2017 Barcelona Marathon, 23 Caucasian non-elite runners were voluntarily recruited through the event’s organizing committee; four of them did not finish the marathon and thus did not complete the study. The participants who were under 18 years or over 50 years, along with those of known chronic pathology, were excluded. The sample size of 19 males (41 ± 4 years old; 77.4 ± 7.1 kg; 1.80 ± 0.1 m; 24.0 ± 2.1 kg/m^2^) ran 2.7 ± 1.6 h/training per week and had a running history of 8.2 ± 5.1 years, on average, prior to the race. All of them were weighed, only with running pants on, 2 h before the race, immediately after completing the race, and at 48 h after the race using the same scale (Jata 565, Barcelona, Spain). Core temperature was measured using a tympanic thermometer (Braun ThermoScan 7—IRT6520).

The Barcelona Marathon 2017 (42.195 km on asphalt at sea level) began at 8:30 h at a temperature of 11–14°C and 68–79% humidity. During the race, the runners were provided with guidelines to maintain adequate levels of hydration by eating and drinking at aid stations every 5 km, with such sustenance as mineral water, sports drinks, fruits, and nuts. The first liquid intake was programmed at 60 min into the race: 400 ml for lighter/slower runners and 800 ml for heavier/faster runners, drinking 100–150 ml every 15–20 min. Commercialized beverages were provided to runners with an average of 480 mg/l for Na^+^, 85 mg/l for K^+^, and 45 mg/l for Mg^2+^. The race time recorded of each participant was the official time from the race organization.

Ethics approval was obtained from the Hospital Universitari Germans Trias i Pujol (ICOR-2017-04, REF.CEI, PI-17-037) according to the principles of the Helsinki Declaration for experiments with human beings. All the participants gave signed informed consent.

### Clinical Data

Data on serum and urine biomarkers of kidney function (serum creatinine, urea, serum sodium, and urine test strip), inflammation (C-reactive protein), and muscle damage (creatine kinase) were collected 24 h pre-race, immediately post-race, and at 48 h post-race.

Fasting blood samples (3 × 10 ml) were obtained from the antecubital vein in EDTA vacutainers at 24 h pre-race, immediately post-race, and at 48 h post-race. The blood samples were centrifuged at 3,000 rpm at 4°C for 10 min in a bench-top centrifuge. The supernatant serum was aliquoted and stored on dry ice until all samples were frozen at −80°C in sealed Eppendorf tubes to avoid evaporation. Serum urea, creatinine, CRP, CK, and Na^+^ were determined using an AU-5800 Chemistry Analyzer (Beckman Coulter Inc., CA, United States). Serum osmolality was determined with an Advanced Micro-Osmometer, Model 3300 (Advanced Instruments Inc., MA, United States) using freezing point depression. Urine tests were measured with AUTION MAX AX-4030 (ARKRAY Inc., MN, United States) analyzer with Uriflet S 9UB strips.

### Whole-Body Bioimpedance Measurements

**FIGURE 1:**
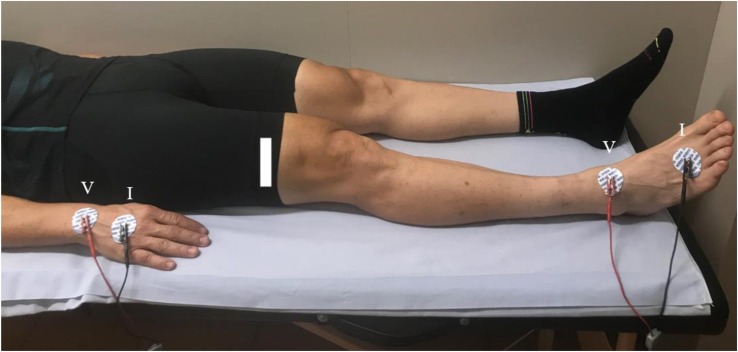
The standard distal BIA configuration electrode placement.

Tetra-polar bioimpedance measurements were taken at 24 h pre-race, immediately post-race, and at 48 h post-race according to the protocol of the standard distal BIA configuration, known as whole-body (Foster and Lukaski, 1996), with the participant in supine decubitus. Four adhesive contact electrodes Ag/AgCl (COVIDIEN Ref. 31050522, COVIDIEN LLC, Mansfield, IL, United States)—two for injection of current (I) and two for detecting voltage (V)—were dorsally placed on the right hand in the third metacarpo-phalangeal articulation and in the carpus, respectively, 5 cm apart. The pair on the foot was located in the third metatarso-phalangeal and in the articulation, 6 cm apart (Figure 1).

The measurements were obtained at 50 kHz with a phase-sensitive bioimpedance analyzer (BIA-101 Anniversary; AKERN-Srl, Florence, Italy), which injected a constant, sinusoidal alternating current of 245 μARMS. The measurement or technical errors of the system, determined with a parallel circuit of precision resistor and capacitor, were <1 Ω for R and <2% for capacitance.

The supporting staff used a towel to remove dirt and sweat before the electrode application. The measurement was done 15 min immediately post-race, with a core temperature at around 36°C. Inside the sanitary facility, the temperature was 21–24.5°C and the humidity was 36–38%.

### Statistical Analysis

Data were collected at 24 h pre-race, immediately post-race, and at 48 h post-race, considering the values taken at 24 h pre-race as baseline. The normality of distribution of the variables was checked by the Shapiro–Wilk test and the homogeneity of variances by Levene’s test.

Repeated-measures ANOVA test was used to determine the effect of the marathon on variables measured at 24 h pre-race, immediately post-race, and at 48 h post-race using the multiple-comparison Bonferroni tests. Friedman test was used for repeated measure of data non-normally distributed with the Wilcoxon test.

In addition, the samples were separated in two groups according to the cutoff of ≥1.2 mg/dl of serum creatinine post-race (G1, serum creatinine post-race <1.2 mg/dl and G2, serum creatinine post-race ≥1.2 mg/dl) considering normal values of 0.6–1.2 mg/dl by the Clinical Laboratory of the Hospital Universitari Germans Trias i Pujol (AU-5800 Chemistry Analyzer (Beckman Coulter Inc; California, United States). Creatinine clearance rate (CCr or CrCl) is a useful measure for approximating the GFR (Stevens and Levey, 2009). It was calculated according to the Cockcroft–Gault equation (Cockcroft and Gault, 1976) and taking into account that CCr <60 ml/min/1.73 m^2^ precedes the onset of kidney failure (Kdigo., 2013). Mann–Whitney’s U test and Student’s *t*-test were applied to analyze non-parametric and parametric variables, respectively.

The variables normally distributed are shown as mean ± SD, while the non-normally distributed data are shown as median and interquartile range (IQR). The level of statistical significance was set at *P* < 0.05. The statistical software IBM^®^ SPSS^®^ version 24.0 (Armonk, NY: IBM Corp., United States) was used for data analysis.

### Bioelectrical Impedance Vector Analysis

In this study, the individual bioimpedance vectors of runners [normalized by the runner’s height (H) R/H, Xc/H, and PA] measured at 24 h pre-race, immediately post-race, and at 48 h post-race were plotted on the tolerance ellipses (RXc-graph) of the Caucasian ethnicity adult population (Piccoli et al., 1995) by using the BIVA software (Piccoli and Pastori, 2002).

The differences in the mean bioimpedance vectors obtained at 24 h pre-race and immediately post-race were plotted with their 95% confidence on the dRXc-graph. The paired one-sample Hotelling’s T2 test (Hotelling, 1931), a multivariate extension of Student’s t-test for paired data, was used to compare the differences of the mean bioimpedance vectors. If a 95% confidence ellipse of a vector displacement covers the null vector (*i*.*e*., abscissa 0, ordinate 0), the vector displacement is not statistically significant (*P* < 0.05) at a paired one-sample Hotelling’s T2 test.

The two-sample Hotelling’s T2 test, a multivariate extension of Student’s t-test for unpaired data, was used to compare the mean vectors from two groups (G1 and G2) according to the cutoff of ≥1.2 mg/dl serum creatinine value immediately post-race. If the 95% confidence intervals of group mean do not overlap, the group means are significantly different (*P* < 0.05), but the reverse is not necessarily true. Hotelling’s T2 test is more sensitive than Student’s *t*-test performed on each variable and entails a smaller risk of erroneously rejecting the null hypothesis.

The sample size was estimated for independent groups according to R/H (*n* = 6 and 3 by group) and Xc/H (*n* = 14 and 7 by group) value to have a power (β = 0.80) to detect an effect size of 0.996, with a 0.05 significance level using MedCalc statistical software version 19.0.3 (MedCalc Software bvba, Ostend, Belgium).

In addition, the area under the receiver operating characteristic (ROC) curve was used to measure the discriminative capacity of R/H, Xc/H, and PA immediately post-race and at 48 h post-race in a clinical situation of AKI stage 1 according to the cutoff of ≥1.2 mg/dl of serum creatinine post-race.

## Results

Table 1 shows the mean ± SD values of body weight, body mass index (BMI), race time, and percentage change of body weight as well as the result of the statistical analysis. A decrease of body weight is related to a negative percentage change of body weight, while an increment of body weight is related to a positive percentage change with respect to the baseline values.

**TABLE 1:** Anthropometric and training data of 19 non-elite marathon runners.

Parameters	24 h pre-race (1)	Immediately post-race (2)	48 h post-race (3)	*F P value^a^*	*SE P value1-2^b^*	*SE P value1-3^b^*	*SE P value2-3^b^*
Weight (kg)	77.4 ± 7.1	75.0 ± 7.0	78.1 ± 7.1	79.691 0.000	0.285 0.000	0.233 0.492	0.219 0.000
BMI (kg m^–2^)	24.0 ± 2.1	23.2 ± 2.0	24.2 ± 2.1	75.945 0.000	0.092 0.000	0.073 0.0561	0.070 0.000
Difference Weight (%)^2–1^	−3.2 ± 1.5		–	–	–	–	–
Difference Weight (%)^3–1^	–	0.5 ± 1.3		–	–	–	–
Race time (hh:mm:ss)	3:33:00 ± 0:24:16						

*^*a*^Repeated measures ANOVA test; ^*b*^Bonferroni test. BMI, body mass index.*

The repeated-measures ANOVA test showed statistical significance (*P* < 0.01) for both body weight and BMI. The multiple-comparison Bonferroni tests showed significant differences (*P* < 0.01) immediately post-race compared to 24 h pre-race and immediately post-race compared to 48 h post-race for body weight and BMI values. However, no statistical significance (*P* > 0.05) was found between values in body weight and BMI at 24 h pre-race and at 48 h post-race. Among the runners, 63.2% recovered their body weight baseline values at 48 h post-race.

### Clinical Data and BIVA at 24 h Pre-race, Immediately Post-race, and at 48 h Post-race

Table 2 shows the clinical data measured at 24 h pre-race, immediately post-race, and at 48 h post-race as mean ± SD for normally distributed variables and as median (IQR) for non-normally distributed data. In addition, also presented are the results of repeated-measures ANOVA test using multiple-comparison Bonferroni tests and of Friedman test with Wilcoxon test for data which were non-normally distributed.

**TABLE 2:** Serum and urine biomarkers of 19 non-elite marathon runners.

Parameters (normal reference values)	24 h pre-race (1)	Immediately post-race (2)	48 h post-race (3)	*F P value^a^*	*SE P value1-2^b^*	*SE P value1-3^b^*	*SE P value2-3^b^*
Serum Creatinine (mg/dL) (0.6–1.2 mg/dL)	0.96 ± 0.09	1.28 ± 0.20	0.91 ± 0.09	46.491 0.000	0.039 0.000	0.013 0.014	0.038 0.000
Serum Urea (mg/dL) (17–43 mg/dL)	35.2 ± 8.9	43.2 ± 9.4	35.2 ± 6.9	10.326 0.001	1.919 0.002	1.496 1.000	1.851 0.001
Serum Sodium (mmol/L) (135–145 mmol/L)	139.3 ± 1.1	141.6 ± 3.0	139.2 ± 1.5	6.214 0.009	0.720 0.010	0.449 1.000	0.716 0.007
Serum Osmolality (mmol/kg) (285–295 mmol/kg)	287.0 ± 3.9	292.7 ± 6.6	285.2 ± 2.2	14.109 0.000	1.488 0.001	1.488 0.663	1.488 0.000

**Parameters (normal reference values)**	**24 h pre-race (1)**	**Immediately post-race (2)**	**48 h post-race (3)**	***Chi-square P value^c^***	***Z P value2-1^d^***	***Z P value3-1^d^***	***Z P value3-2^d^***

Serum CRP (mg/L) (0–5.0 mg/L)	0.70 (0.20–5.30)	0.50 (0.20–3.00)	7.50 (3.20–15.50)	30.778 0.000	−1.880 0.050	−3.824 0.000	−3.825 0.000
Serum CK (U/L) (20–215 U/L)	140 (76–400)	376 (260–946)	685 (194–1882)	31.684 0.000	−3.823 0.000	−3.823 0.000	−3.099 0.000
Hematuria	0	1 case	0	–	–	–	–
Proteinuria	0	5 cases	0	–	–	–	–

*^*a*^Repeated measures ANOVA test; ^*b*^Bonferroni test; ^*c*^Friedman test with Chi-square; ^*d*^Wilcoxon test. CRP, C-reactive protein; CK, creatine kinase.*

A repeated test showed statistical significance (*P* < 0.01) in serum creatinine, serum urea, serum sodium, C-reactive protein, and creatine kinase.

The serum urea post-race values increased significantly (*P* < 0.01) compared to baseline, and 10 of 19 runners (53%) were above 43 mg/dl. Serum creatinine increased significantly post-race compared to baseline (*P* < 0.01), and 12 of 19 runners (63%) had values over the cutoff of ≥1.2 mg/dl. The CRP at 48 h post-race increased significantly (*P* < 0.01) compared to baseline, and 13 of 19 runners (68%) showed values above 5.0 mg/L (5.2–15.5 mg/L), with an increase of more than 800% of their baseline values. The CK at 48 h post-race increased significantly (*P* < 0.01) compared to baseline, and 18 of 19 runners (95%) showed values above 215 U/L (256–1,882 U/L), with an increase from 70 to 1,500% of their baseline values. Any CK values at 48 h post-race were greater than 2,000 U/L.

The sodium serum levels also increased significantly immediately post-race with respect to baseline (*P* < 0.01), but within normal reference values; however, only two of 19 runners (10%) had sodium serum levels above 145 mmol/L post-race.

The osmolality serum levels also increased significantly immediately post-race with respect to baseline (*P* < 0.01), but within normal reference values (285–295 mmol/kg), except in four of 19 runners (21%) who had levels above 295 mmol/kg post-race.

Regarding the urine test, there were some cases of hematuria (one of 19 runners, 5%) and proteinuria (five of 19 runners, 25%) found only after the race.

Table 3 shows the values of R/H, Xc/H, and PA described as mean ± SD, the Pearson’s correlation coefficient, r, between R/H and Xc/H, and the result of repeated-measures ANOVA test using multiple-comparison Bonferroni tests.

**TABLE 3:** Whole-body bioimpedance measurements of 19 non-elite marathon runners.

Parameters	24 h pre-race (1)	Immediately post-race (2)	48 h post-race (3)	*F P value^a^*	*SE P value1-2^b^*	*SE P value1-3^b^*	*SE P value2-3^b^*
R/H (Ω/m)	271.7 ± 15.3	282.5 ± 19.4	264.6 ± 18.0	23.977 0.000	3.595 0.023	2.740 0.055	2.608 0.000
Xc/H (Ω/m)	31.2 ± 2.8	34.0 ± 3.6	29.6 ± 2.8	41.134 0.000	0.665 0.000	0.368 0.000	0.547 0.001
PA (°)	6.5 ± 0.4	6.9 ± 0.5	6.4 ± 0.5	11.371 0.001	0.121 0.048	0.086 0.218	0.102 0.000
r	0.69	0.79	0.62	–	–	–	–

*^*a*^Repeated measures ANOVA test; ^*b*^Bonferroni test. R/H, resistance/height; Xc/H, reactance/height; PA, phase angle.*

A repeated ANOVA test showed high statistical significance (*P* < 0.01) in R/H and Xc/H as well as PA. Using multiple-comparison Bonferroni tests, Xc/H showed significant differences (*P* < 0.01) immediately post-race compared to values at 24 h pre-race and at 48 h post-race compared to values at 24 h pre-race. In addition, Xc/H showed significant differences (*P* < 0.01) at 48 h post-race compared to immediately post-race. However, R/H and PA only showed significant differences immediately post-race compared to values at 24 h pre-race (*P* < 0.05) and at 48 h post-race compared to the values immediately post-race (*P* < 0.01). Any significant differences were found in R/H and PA at 48 h post-race compared to values at 24 h pre-race (*P* > 0.05).

Using the BIVA software (Piccoli and Pastori, 2002), Figure 2 shows the individual bioimpedance vectors at time points 24 h pre-race, immediately post-race, and 48 h post-race for 19 non-elite marathon runners compared with the tolerance ellipses for healthy Caucasian adults (Piccoli et al., 1995) on RXc-graph. Two runners (runner_11 and runner_17) had a vector displacement immediately post-race outside of the 50% tolerance ellipse for healthy Caucasian adults; however, this reverted to 50% tolerance ellipse at 48 h post-race. Thus, all vector positions were within the range of normal values. Two other runners (runner_14 and runner_15) had a vector displacement at 48 h post-race outside of the inferior pole of the 50% tolerance ellipse for healthy Caucasian adults, indicating an expansion on extracellular water and inflammation.

**FIGURE 2:**
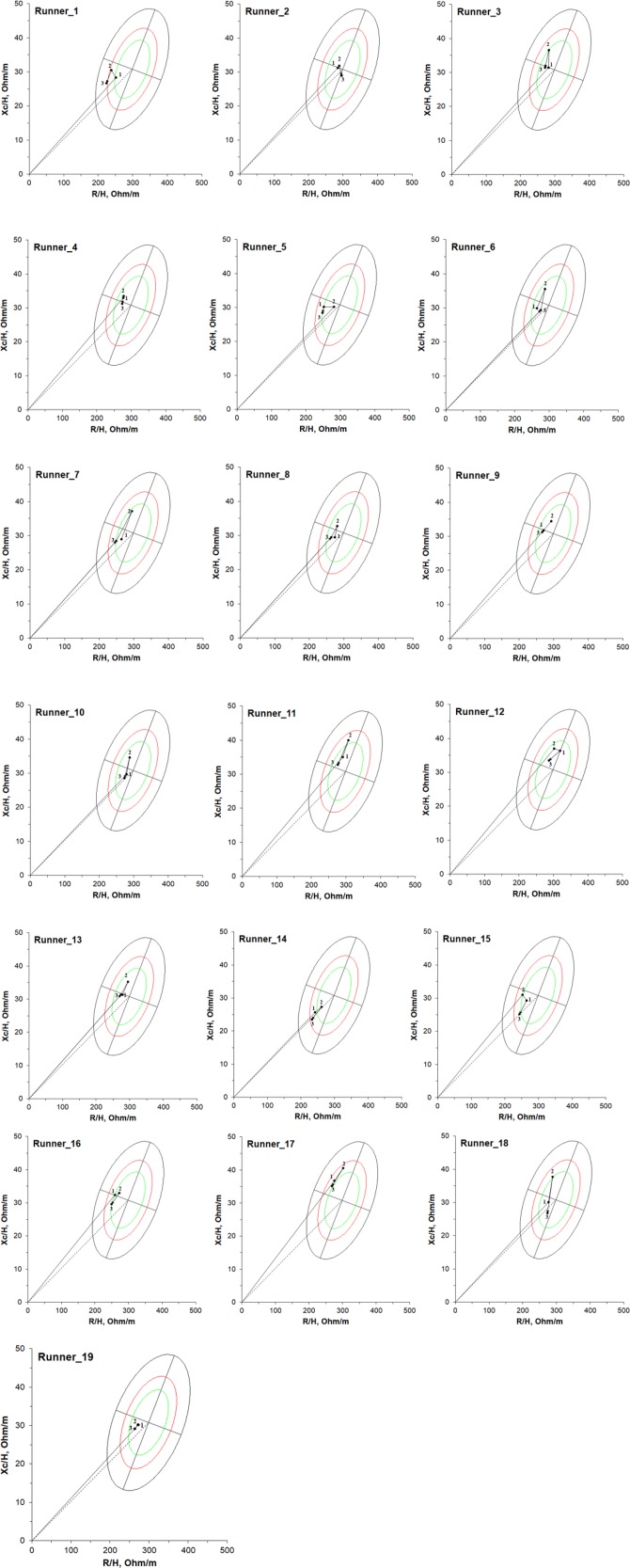
Individual vectors on RXc-graph for 19 non-elite marathon runners 24 h pre-race (1), immediately post-race (2), and 48 h post-race (3); compared to the tolerance ellipses (50%, 75% and 95% percentiles) of the reference population.

**FIGURE 3:**
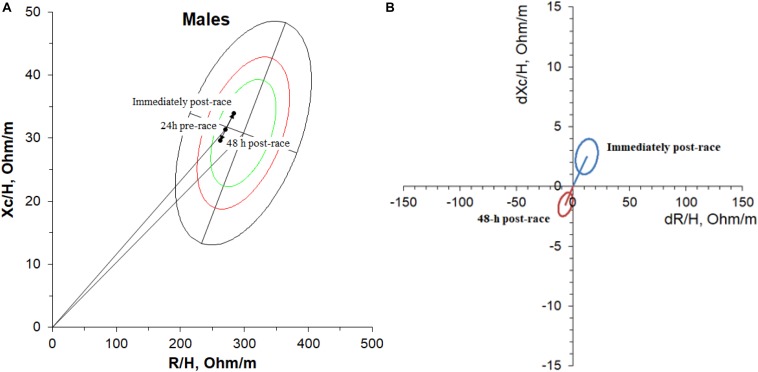
Mean bioimpedance vectors **(A)** 24 h pre-race, immediately post-race, and 48 h post-race on RXc-graph and paired graph **(B)** for 19 non-elite marathon runners. The paired graph shows the 95% confidence ellipse corresponding to the mean bioimpedance vector difference obtained immediately post-race and 48 h post-race compared to baseline (24 h pre-race).

Figure 3A shows the mean bioimpedance vector. The vector displacement and lengthening along the major axis was significant compared to baseline but did not cross through the 75% area of tolerance ellipses of the reference population. The paired graph (Figure 3B) shows the 95% confidence ellipse corresponding to the mean of the difference obtained immediately post-race compared to the vector (baseline) at 24 h pre-race and at 48 h post-race compared to the baseline vector using the BIVA software (Piccoli and Pastori, 2002). The 95% confidence ellipse did not cover the null vector (*i*.*e*., dZ/H = 0.0), indicating a statistically significant vector displacement (*P* < 0.05) at a paired one-sample Hotelling’s T2 test (Hotelling, 1931).

### Clinical Data and BIVA According to the Cutoff of ≥ 1.2 mg/dl of Serum Creatinine Immediately Post-race

Table 4 shows the results of anthropometric and clinical data according to a cutoff of ≥ 1.2 mg/dl of serum creatinine level immediately post-race for G1 (< 1.2 mg/dl of serum creatinine level) and G2 (≥1.2 mg/dl of serum creatinine level) as mean ± SD for normally distributed variables and as median (IQR) for non-normally distributed data. In addition, also presented are the statistical results of Mann–Whitney’s U test or Student’s t-test for non-parametric and parametric variables, respectively.

**TABLE 4:** Anthropometric and clinical data of 19 non-elite marathon runners according to cut-off of ≥1.2 mg/dL of serum creatinine level immediately post-race.

Parameters Immediately post-race	G1, n = 7 (<1.2 mg/dL)	G2, n = 12 (≥1.2 mg/dL)	*P value*
Weight (kg)	70.6 ± 5.7	77.5 ± 6.5	0.035
Weight-lost (%)	−2.83 ± 1.28	−3.44 ± 1.55	0.397
BMI (kgm^–2^)	22.0 ± 1.7	23.9 ± 1.9	0.047
Serum Urea (mg/dL)	40.9 ± 9.2	44.4 ± 9.2	0.429
Serum Creatinine (mg/dL)	1.07 ± 0.07	1.39 ± 0.15	0.000
Increment Serum Creatinine (mg/dL)	0.17 ± 0.04	0.40 ± 0.16	0.000
CCr (mL/min)	93.2 ± 10.1	76.4 ± 9.6	0.002
Serum CRP^§^ (mg/L)	4.40 (2.80–5.50)	7.50 (3.90–8.70)	0.001
Serum CK^§^ (U/L)	389 (194–890)	871 (296–1882)	0.035
Serum Sodium (mmol/L)	142.0 ± 2.2	141.5 ± 3.4	0.730
Osmolality (mmol/kg)	290.1 ± 5.0	294.3 ± 7.1	0.286
Hematuria	0 cases	1 case	–
Proteinuria	0 cases	5 cases	–

*^§^48h post-race values. BMI, body mass index; CCr, creatinine clearance rate; CRP, C-reactive protein; CK, creatine kinase.*

According to the cutoff of ≥ 1.2 mg/dl serum creatinine, 63% of the runners (12 of 19) corresponding to G2 showed an increment of serum creatinine post-race and 58% of them (seven of 12) showed an increment in serum urea post-race of above 43 mg/dl (cutoff value of serum urea). Serum creatinine reached values of AKI stage 1, showing increases of ≥ 0.3 mg/dl in 83% of the runners (10 of 12) and increases of ≥ 0.5 mg/dl in 42% of the runners (five of 12).

The CCr post-race showed a significant decrease (*P* < 0.01) upon comparing G1 and G2, although none of the runners (G1 and G2) showed CCr values below 60 ml/min/1.73m^2^. The CK at 48 h post-race showed a significant increase (*P* < 0.05) upon comparing G1 and G2. All G2 runners had CK values over 215 U/L (282–1,882 U/L). The C-reactive protein at 48 h post-race showed a significant increase (*P* < 0.01) upon comparing G1 and G2. It should be noted that in G2 there are the only cases of hematuria (8%) and proteinuria (42%) among all the runners analyzed. Regarding serum urea, serum sodium, and serum osmolality, no significant differences were seen between the groups (*P* > 0.05).

According to the cutoff of ≥ 1.2 mg/dl serum creatinine, no significant differences were observed in weight loss immediately post-race (*P* > 0.05). G1 showed a weight loss immediately from 1.6 to 4.1% and likewise for G2 from 1.9 to 5.0%. However, significant differences were observed in both body weight and BMI (*P* < 0.05).

Table 5 shows the values of R/H, Xc/H, and PA immediately post-race and at 48 h post-race, described as mean ± SD of G1 and G2. In addition, also presented are the Pearson’s correlation coefficient, r (R/H, Xc/H), and the statistical significance of Student’s t-test for two independent samples.

**TABLE 5:** Whole-body bioimpedance measurements (R/H, Xc/H, and PA) immediately post-race, 48 h post-race and statistical results between G1 and G2, according to cut-off of ≥1.2 mg/dL serum creatinine level immediately post-race.

	G1, *n* = 7 (<1.2 mg/dL)		G2, *n* = 12 (≥1.2 mg/dL)		*P value^2^ (G1 vs. G2)*	*P value^3^ (G1 vs. G2)*
	Immediately post-race (2)	48 h post-race (3)	Immediately post-race (2)	48 h post-race (3)		
R/H (Ω/m)	292.9 ± 14.4	274.1 ± 6.7	276.4 ± 19.8	259.0 ± 8.3	0.072	0.077
Xc/H (Ω/m)	35.6 ± 2.6	31.9 ± 1.9	33.1 ± 3.9	28.2 ± 2.4	0.151	0.003
PA (°)	6.94 ± 0.41	6.64 ± 0.40	6.82 ± 0.49	6.23 ± 0.44	0.609	0.065
r	0.60	0.52	0.65	0.59	–	–

*^2^Immediately post-race G1 vs. G2; ^3^48 h post-race G1 vs. G2. R/H, resistance/height; Xc/H, reactance/height; PA, phase angle.*

No statistical significance was found in whole-body bioimpedance measurements immediately post-race upon comparing G1 *vs*. G2. However, a high statistical significance (*P* < 0.01) was found in Xc/H at 48 h post-race, with a tendency to be statistically significant in R/H and PA.

**FIGURE 4:**
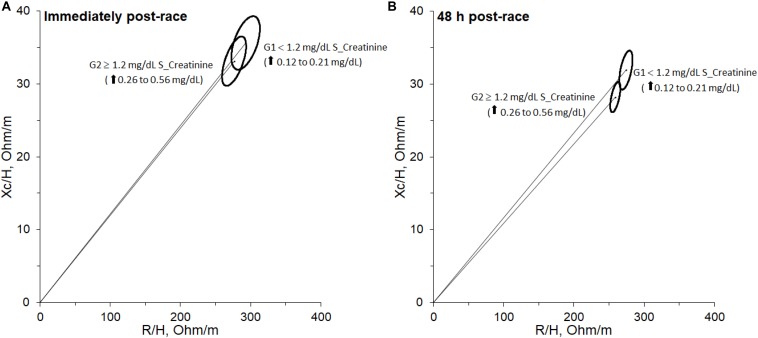
95% confidence ellipse for both groups immediately post-race **(A)** and 48 h post-race **(B)** separated according to cut-off of ≥1.2 mg/dL of serum creatinine level immediately post-race.

Figure 4A shows an overlapping of 95% confidence ellipses between G1 and G2 immediately post-race according to the cutoff of ≥ 1.2 mg/dl serum creatinine level immediately post-race. BIVA method with Hotelling’s T2 test shows no significant differences (T2 = 3.87, *F* = 1.82, *P* > 0.05). However, Figure 4B shows separated 95% confidence ellipses between G1 and G2 at 48 h post-race according to the cutoff of ≥ 1.2 mg/dl serum creatinine level immediately post-race. Through BIVA method with Hotelling’s T2 test, the mean groups are significantly different (T2 = 18.54, *F* = 8.73, *P* < 0.05).

According to Figure 2, the runners that belong to the group G2 are runner_1, runner_2, runner_5, runner_6, runner_7, runner_10, runner_11, runner_14, runner_15, runner_16, runner_18, and runner_19.

**FIGURE 5:**
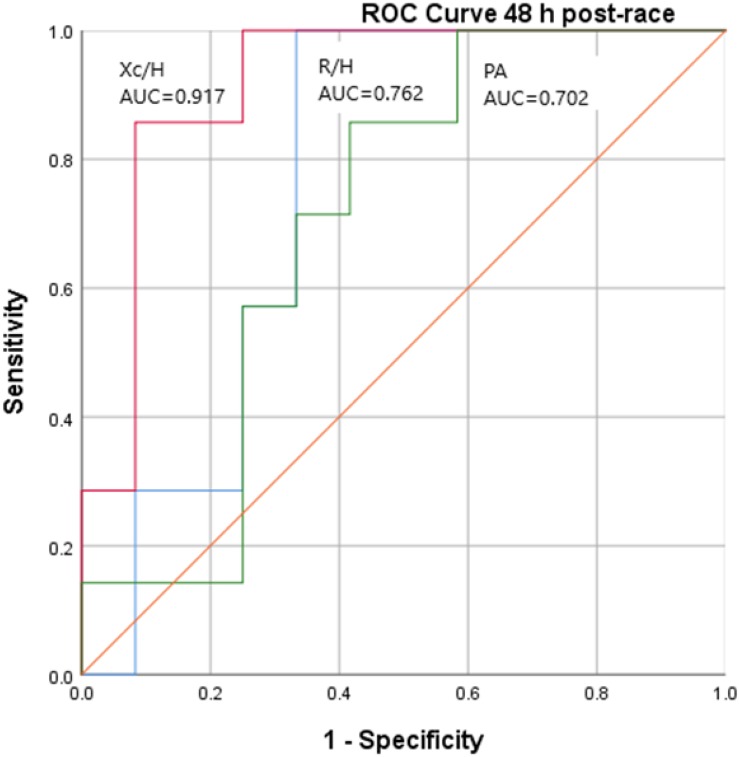
ROC Curve of R/H, Xc/H, and PA 48 h post-race according to cut-off of ≥1.2 mg/dL of serum creatinine post-race.

Figure 5 shows the area under the ROC curve of R/H, Xc/H, and PA at 48 h post-race in the clinical situation of AKI stage 1 according to the cutoff of ≥ 1.2 mg/dl serum creatinine post-race.

According to AKI stage 1, the Xc/H at 48 h post-race variable shows a high significant difference (*P* = 0.003), with area below the ROC curve of 0.917 and a standard error of 0.067.

## Discussion

This study analyzes the relationship between whole-body bioimpedance vector displacement, by BIVA, and renal function through serum and urine biomarkers, after a marathon, in non-elite marathon runners.

Transient significant increases of urea and creatinine, indicating renal dysfunction, have been described in runners after completion of the marathon (Kratz et al., 2002; McCullough et al., 2011). A repeated-measures test showed high statistical significance (*P* < 0.01) in serum creatinine, urea, serum sodium, CRP, and CK. Using multiple-comparison tests, serum creatinine showed statistical significance (*P* < 0.01) immediately post-race and at 48 h post-race with respect to the values at 24 h pre-race and at 48 h post-race with respect to the values immediately post-race. The inflammation biomarker CRP and the muscle damage biomarker CK showed significantly different values (*P* < 0.01) at 48 h post-race compared to baseline values according to its rate of production, with concentration peaks at 48 h. The mean bioimpedance vector showed a lengthening along the major axis, with a migration above (left) the minor axis of the tolerance ellipse, immediately post-race compared to baseline, relating to the fluid loss and muscle activation during a marathon. A 50-kHz, phase-sensitive BIA analyzer yields passive bioelectrical measurements of bioimpedance module (Z) and its components R (which is the opposition to the flow of an alternating current through intra- and extra-cellular ionic solutions) and Xc (which indicates the capacitive component of tissue interfaces and cell membranes). The parameter Xc is associated with the integrity of the soft tissue structures and R with the hydration state of the soft tissue. The phase angle (PA), calculated as the arc tangent of Xc/R, allows the non-invasive assessment of intra- to extracellular fluid (Lukaski et al., 2019). Changes in bioimpedance variables are indicators of fluid (R) and cell structure integrity (Xc and PA). Muscle contraction can be described based on two variables, length and tension, which imply a major delay between I and V and, as consequence, major Xc and PA post-race.

The paired graph (mean of the difference post-race compared to the baseline vector) did not cover the null vector (*i*.*e*., dZ/H = 0.0), indicating a statistically significant vector displacement (*P* < 0.05) at a paired one-sample Hotelling’s T2 test. Although the mean differences at 48 h post-race compared to the baseline vector did not cover the null vector, there is a tendency to cover it (recovering the pre-race values).

Several factors induce transient changes on renal filtration function, such as hemodynamic influences on renal blood flow, neurohormonal, inflammatory, or oxidative stress factors (McCullough et al., 2011). In the current study, the subjects who developed AKI stage 1 (G2) had more CK and more CRP at 48 h post-race than the group which did not develop AKI stage 1. G2 showed an increase of CK at 48 h post-race by threefold to 16-fold above the reference values measured at 24 h pre-race, while G1 showed an increase of one- to sixfold above the reference values. Although no runners presented CK over 1,890 U/L at 48 h post-race (ninefold above the normal reference values), not close to exertional (exercise-induced) rhabdomyolysis (Kenney et al., 2012), we believe that the inflammation situation that occurs, together with other factors that may be intra-renal or thermal hemodynamic, may be the cause of the kidney damage (Mansour et al., 2017). Although it is not known which renal structure is damaged, there are data that suggest that the tubule may be damaged (Mansour et al., 2017). The alterations in the urinary sediment and the urinalysis observed (hematuria and proteinuria) are less described and also certify the involvement of renal parenchyma. To date, an increased risk of chronic renal failure in marathoners has not been described, but we cannot deny that repeated kidney damage may have long-term negative effects. AKI stage 1 showed a significant Spearman correlation with CRP values at 48 h post-race (*r* = 0.767, *P* = 0.000) and with CCr immediately post-race (*r* = −0.869, *P* = 0.000). However, AKI stage 1 shows no correlation with CK at 48 h post-race (*r* = 0.376, *P* = 0.113). Exertional rhabdomyolysis can be a complication presenting as asymptomatic physiologic elevations of creatine kinase after exercise, leading to acute kidney injury (Szczepanik et al., 2014), but none of the non-elite runners analyzed presented with rhabdomyolysis. This could indicate that due to the fact that marathon does not involve eccentric contractions, rhabdomyolysis is less common (Clarkson, 2007).

Previous studies have related the effects of endurance exercise to serum C-reactive protein and inflammatory markers (Kasapis and Thompson, 2005). All runners analyzed had increases in CRP of 190–7,200% at 48 h after the race compared to the reference levels, showing that global inflammation may be related to the inflammation of the nephron (correlated with a significant increase of creatinine levels).

On the other hand, G2 showed a significant increase (*P* < 0.05) in CRP values at 48 h post-race (290–7000%) compared to baseline (24 h pre-race). Among G2 runners, 92% showed very high levels of CRP values over the cutoff (> 5 mg/L), which is associated with an independent predictor of AKI (Tang et al., 2018), and 83% of them showed high increase levels of creatinine (≥ 0.3 mg/dl), which is associated with AKI stage 1 (McCullough et al., 2011). Thus, the transient reduction in renal filtration function is more related to inflammatory factors (CRP, *P* < 0.01) than to muscle damage (CK, *P* < 0.05).

According to the cutoff of ≥ 1.2 mg/dl serum creatinine, no significant differences were observed in weight loss immediately after the race (*P* > 0.05) between G1 and G2. After a marathon, body weight loss is at around 2–3% (Sawka et al., 2007; Thomas et al., 2016) and at 5–6% after ultra-endurance events without clinical implications (Tam and Noakes, 2013). G1 showed a weight loss of 2–4% and G2 of 2–5% without renal implications.

According to the cutoff of ≥ 1.2 mg/dl serum creatinine post-race, neither has there been significant differences (*P* > 0.05) between G1 and G2 in terms of serum osmolality post-race values used to evaluate the hemoconcentration influences on renal blood flow.

By BIVA, the 95% of confidence ellipses at 48 h post-race showed significant differences (T2 = 18.54, *F* = 8.73, *P* < 0.05) between runners with serum creatinine levels post-race of ≥ 1.2 mg/dl (G2) compared to those with serum creatinine levels < 1.2 mg/dl (G1). This significant difference is mainly due to the parameter Xc/H (*P* < 0.01), which showed a decrease in G2 at 48 h post-race with respect to values at 24 h pre-race from -3.3 to -12.4% and in G1 from 0 to -3.1%. According to the BIVA method (Piccoli et al., 1994), this decrease is due to the expansion of extracellular water (fluid overload) and inflammation. The Xc/H at 48 h post-race showed a significant Spearman rho correlation with CRP at 48 h post-race (*r* = −0.467, *P* = 0.044). AKI stage 1 showed a significant Pearson correlation with Xc/H at 48 h post-race (*r* = − 0.710, *P* = 0.001) and phase-angle (PA) at 48 h post-race (*r* = − 0.586, *P* = 0.008). In addition, among R/H, Xc/H, and PA at 48 h post-race, the variable with major area under ROC curve was Xc/H 48h post-race (AUC = 0.917). The runners with serum creatinine levels post-race ≥ 1.2 mg/dl (G2) showed Xc/H at 48 h post-race values ≤ 30.5 Ω, with 85.7% sensitivity and 91.7% specificity.

The major limitation of this study is the small sample size and the fact that only the male gender was analyzed. When performing the BIVA method, the male and the female samples cannot be processed together because of the reference values (R and Xc) of the healthy population which change according to gender (male and female) and age range (childhood and adults). For this reason, we found the tolerance ellipses (50, 75, and 95%) of the reference population to beseparated by gender (Piccoli et al., 1995). However, the strength of this study is the use of a non-invasive method (BIVA) to assess renal function and muscle damage.

## Conclusion

The transient reduction in renal filtration function is more related to the inflammatory factors rather than to muscle damage. In all the runners observed, serum creatinine and urea values at 48 h post-race compared to baseline values were recovered. There is a direct relationship between a marathon race in non-elite runners and the serum biomarkers of kidney function and inflammation, with a high percentage of AKI stage 1. The runners with AKI stage 1 presented greater CRP values at 48 h post-race, which is associated with systemic inflammation. Phase-sensitive measurements at 50 kHz with BIVA could be a non-invasive method to assess kidney function in runners through the Xc/H value at 48 h post-race, with a decrease of > 3% with respect to the baseline value. It would be necessary to study several consecutive marathon races, adding 24 h post-race measurements, on runners who frequently suffer transient AKI stage 1, including female runners, to determine the long-term negative effects on renal function.

## Data Availability Statement

The datasets generated for this study are available on request to the corresponding author.

## Ethics Statement

Ethics approval was obtained from the Hospital Universitari Germans Trias i Pujol (ICOR-2017-04, REF.CEI, PI-17-037) according to principles of the Helsinki Declaration for experiments with human beings. The patients/participants provided their written informed consent to participate in this study.

## Author Contributions

LN, ER, PB-F, JH-H, AB-G, and JA designed the experiments, analyzed the data, revised the manuscript, and approved the final version of the manuscript. LN, processed the data and prepared the tables and figures. LN and ER, drafted the manuscript.

## Conflict of Interest

The authors declare that the research was conducted in the absence of any commercial or financial relationships that could be construed as a potential conflict of interest.
